# Depressive and Anxious Temperaments as Predictors of Late Onset Bipolar Disorder? Preliminary Results of a “Real World” Exploratory Study

**DOI:** 10.3389/fpsyt.2022.836187

**Published:** 2022-02-17

**Authors:** Laura Orsolini, Giulia Menculini, Silvia Tempia Valenta, Michele Fiorani, David Rocchetti, Virginio Salvi, Alfonso Tortorella, Umberto Volpe

**Affiliations:** ^1^Unit of Clinical Psychiatry, Department of Neurosciences/DIMSC, School of Medicine and Surgery, Polytechnic University of Marche, Ancona, Italy; ^2^Department of Psychiatry, University of Perugia, Perugia, Italy; ^3^Unit of Clinical Psychiatry, Azienda Ospedaliero Universitaria “Ospedali Riuniti”, Ancona, Italy

**Keywords:** affective temperament, bipolar disorder, late onset bipolar disorder, late onset, late mania, psychogeriatric, temperament

## Abstract

**Introduction:**

Bipolar disorder (BD) onset typically occurs between 15 and 30 years, being diagnosed under the age of 50 in 90% of cases, named “non-late onset BD” (non-LOBD). However, clinical observation of late-onset BD (LOBD) raised some concern regarding a differential psychopathological pattern, outcomes and treatment, including a specific affective temperament vulnerability. Therefore, an exploratory study in the “real world” was carried out by investigating psychopathological and temperamental features of a psychogeriatric cohort of LOBD and non-LOBD subjects.

**Methods:**

A total of 180 patients affected with BD-I, BD-II, and Cyclothymic Disorder were screened in a Mood Disorder Outpatient Service, during the timeframe January 2019-August 2021. Out of 78 enrolled outpatients, 66 (33 non-LOBD, 33 LOBD) were recruited, by the retrospective collection of sociodemographic, cognitive, psychopathological and clinical assessment, including the short-version of the Temperament Evaluation of Memphis, Pisa, and San Diego (TEMPS-M).

**Results:**

LOBD is significantly associated with higher rates of BD-II diagnosis (χ^2^ = 27.692, *p* < 0.001), depressive episodes (*p* = 0.025), mixed states (*p* = 0.009), predominant depressive and anxious affective temperaments (*p* < 0.001). Non-LOBD is significantly associated with higher endocrinological (χ^2^ = 6.988, *p* = 0.008) and metabolic comorbidity (χ^2^ = 5.987, *p* = 0.014), a diagnosis of BD-I, manic episodes, and predominant hyperthymic affective temperaments (*p* = 0.001). GDS (*p* < 0.001) and MSRS (*p* = 0.005) scores were significantly higher in LOBD.

**Conclusion:**

Further longitudinal studies with larger sample sizes and a control group are needed to determine whether LOBD may represent a distinct psychopathological entity from non-LOBD and evaluate differences (if any) in terms of prognosis and treatment between non-LOBD and LOBD.

## Introduction

Life expectancy has considerably increased in the last century due to improved health care, socioeconomic progress, technological and lifestyle changes (1, 2). The World Health Organization (WHO) (3) estimates that by 2050, the worldwide elderly population will increase more than 2-fold, becoming around 22% of the entire population (3). However, the increasing life expectancy generated a profound change in the epidemiology of various diseases, including psychiatric disorders (3, 4).

Psychogeriatrics represents a new emerging neuroscientific branch of psychiatry, born from the above-mentioned social upheaval, following the model of healthy aging to differentiate regular aging-related changes from neurological or psychiatric dysfunctions (5). A psychiatric disease may often have an onset during adolescence or young adulthood, even though the possibility of later onset due to age-specific psychopathological triggers has also been documented (e.g., deterioration of mental and/or physical status, lifestyle and social role changes, isolation and loneliness, and so forth) (6).

Although most cases of bipolar disorder (BD) usually occur before the age of 25, being diagnosed under the age of 50 in 90% of cases [i.e., “non-late onset BD” (non-LOBD)], the onset of BD may potentially happen at all stages of life, from childhood [e.g., very-early-onset BD (VEOBD) and early-onset BD (EOBD)], intermediate/conventional-onset BD to geriatric age [e.g., late-onset BD (LOBD)] (7–10). Scientific literature on LOBD is still scarce. However, it has been hypothesized that rising life expectancy may potentially lead to an overall increased incidence of mood disorders, including BD, in advanced ages (11). Accordingly, one could argue whether non-LOBD and LOBD in the psychogeriatric population could be distinct nosological entities, with different etiopathogenesis, psychopathology, clinical course, and treatment. A well-established hypothesis is that predominant temperament may be a vulnerability factor in the development of BD and may influence the psychopathological manifestation, clinical course, and onset age among BD patients (12–14). In particular, predominant hyperthymic and depressive temperaments have been found to be more related to the “classic” BD picture, while cyclothymic, anxious, and irritable temperaments have been found to be more likely associated with more complex or atypical BD pictures (14). Moreover, the neuroprogression/neurodegenerative BD hypothesis and staging models of BD, as well as the acknowledgment of significant heterogeneity existing between non-LOBD vs. LOBD presentations of the illness (15) might support the potential detrimental role of BD relapses in explaining the differences between non-LOBD and LOBD in the psychopathological course, outcomes, and prognosis, even though data are still contradictory (7, 16). Furthermore, subsyndromal/attenuated symptomatology (i.e., mood symptoms present at a level of intensity below the threshold required to diagnose a mood episode over the life) may dominate the clinical course of BD (17, 18). In particular, it has been already documented that LOBD subjects may experience a burden of episodic and subsyndromal symptomatology (19–21). Therefore, some authors argued whether LOBD may be the expression of an attenuated/subsyndromal vulnerability to BD, triggered by age-specific psychopathological factors (22).

Therefore, due to the poor literature regarding the clinical characterization of LOBD vs. non-LOBD in psychogeriatric BD populations, there is the need for further clinical discrimination between non-LOBD and LOBD presentations, particularly by deepening the affective temperament profile as well as the “organic” and “subsyndromal/attenuated” hypothesis. Moreover, while a clear cut-off age discriminating between non-LOBD and LOBD appears not significant from a clinical standpoint, essentially due to several confounding factors, existing evidence suggests a grossly bi-modal distribution for the age of BD onset, being 50 years old considered a reliable threshold, as already supported by several authors and proposed by the International Society of Bipolar Disorder (ISBD) Task Force (7, 23–27).

Therefore, in the present exploratory study, we aimed at characterizing and comparing non-LOBD and LOBD from a clinical and psychopathological perspective in a cohort of psychogeriatric outpatients with a diagnosis of BD type I (BD-I), type II (BD-II), and Cyclothymic Disorder (CYC). The primary aim was to identify the differences (if any) between non-LOBD and LOBD in basic affective temperamental profiles and their association with a specific psychopathological pattern in later life. Secondary exploratory objectives included investigating whether: (a) psychogeriatric patients with a LOBD may be more likely accompanied by a mild cognitive impairment (MCI) when compared to non-LOBD, by supporting the hypothesis that a LOBD may be a secondary manifestation of cognitive deterioration (“organic hypothesis”); (b) LOBD may represent an attenuated form of the BD spectrum, not diagnosed in early life, due to an attenuated/sub-clinical manifestation, by exploring all variables of the clinical history of recruited patients.

## Materials and Methods

### Study Design and Participants

An exploratory, naturalistic, observational, cross-sectional study was carried out by retrospectively collecting information documented from outpatients' electronic medical records (EMRs) at the Unit of Clinical Psychiatry, Outpatient Service on Mood Disorders, Department of Neurosciences/DIMSC, University Hospital Ospedali Riuniti in Ancona, Italy. At the moment of the first psychiatric consultation, all outpatients were asked to voluntarily provide written consent to use the clinical information collected during their first and follow-up visits for research purposes. Using the information from their EMRs, we retrospectively screened all outpatients aged ≥ 50 years old, consecutively referring to the Mood Disorders outpatient service from January 2019 to August 2021. We selected patients using the following inclusion criteria: (a) age ≥50 years old at the time of assessment; (b) diagnosis of BD-I and BD-II, or CYC, according to the DSM-5 criteria (28); (c) absence of a moderate-to-severe cognitive impairment or dementia and/or with a brain CT and/or MRI indicative of moderate-to-severe cognitive impairment, (d) absence of unipolar major depression, schizoaffective disorder, or other psychotic disorders except for psychotic symptoms related to the current episode of BD; e) absence of current or recent (in the previous 6 months) alcohol and/or substance use disorder, according to the DSM-5 (28); (f) consent to participate in the study and written informed consent to use their data for research purposes. Exclusion criteria were lack of willingness or capacity to provide informed consent to participate in the study.

The study was approved by the local Institutional Review Board and conducted in accordance with the ethical principles outlined in the Declaration of Helsinki and according to the guidelines for Good Clinical Practice (GCP).

### Procedures and Measures

This study used only EMR variables that clinicians collected within standard psychiatric consultation and follow-up visits. The assessment was performed during a direct interview or through the clinical database consultation, leading to the compilation of an *ad hoc* case report form (CRF) for each subject. The CRF included sociodemographic data, recent and past medical history, current and/or past use of substances and/or alcohol, age of onset, clinical course, and the type of BD diagnosis, including the type of affective episode and its specifier at the time of the assessment, history of previous and/or current suicidal and/or self-injurious ideation and/or behaviors and their frequency (total number of lifetime episodes), concomitant medical comorbidities as well as concomitant pharmacological and psychopharmacological treatments.

The diagnosis was made through the MINI-5 clinical interview (Mini-International Neuropsychiatric Interview, Italian translation, version 7.0.0), and only patients who met DSM-5 criteria for BD-I, BD-II, or CYC, were included in the study. Study participants were also divided into two categories according to the age of illness onset: non-LOBD (age of illness onset <50 years old) and LOBD (age of illness onset ≥ 50 years old). The Montreal Cognitive Assessment [MOCA; (29)] was used to screen the sample for possible moderate-to-severe cognitive impairment by measuring visuospatial and executive ability, object naming, memory, attention, language, abstraction, and orientation. Moreover, according to the routinely internal protocol of our outpatient service on Mood Disorders, in those patients with a Montreal Cognitive Assessment (MOCA) total score below 19 and/or with a suspected cognitive deterioration, according to the clinical examination, a brain CT scan and/or a brain magnetic resonance imaging (MRI) is usually performed. Those subjects with a MOCA total score below 19 were excluded in the present study. Clinical status during the interview was evaluated using the Clinical Global Impressions-Bipolar Disorder scale [CGI-BD; (30)]. Functioning was evaluated with the Global Assessment of Functioning scale [GAF; (31)]. The global psychopathology was assessed through the Brief Psychiatric Rating Scale Expanded Version 4.0 [BPRS; (32)], investigating the severity of the disorder in the area of affectivity, negative symptoms, positive symptoms, activity, and disorientation. The Hamilton Depression Rating Scale [HAM-D; (33)] and the Geriatric Depression Scale [GDS; (34)] were used to assess the severity of the depressive episode. The Young Mania Rating Scale [YMRS; (35)], was used to assess manic symptoms; and the Mixed States Rating Scale [MSRS; (36)], to assess mixed states associated with the current affective episode.

All patients filled in the Italian validated short version of the Temperament Evaluation of the Memphis, Pisa, Paris and San Diego [short TEMPS-M; (37)], a 35 items questionnaire used to assess affective temperaments described by Akiskal (depressive, anxious, hyperthymic, cyclothymic and irritable) using a dimensional approach with a five-point Likert type scale ranging from 1 to 5 (1 = “not at all”; 2 = “a little”; 3 = “moderately”; 4 = “much”; 5 “very much”). TEMPS-M displays a good internal consistency (Cronbach α ranging from 0.69 to 0.84) and test-retest reliability. TEMPS-M was used to measure the primary outcome of our study, as it was assumed that the affective disposition could play a key role in the onset, development and clinical course of mood disorders (38).

### Statistical Analysis

Data analysis was performed using Statistical Package for Social Science for MacOS (SPSS) software, Version 27.0 (IBM Corp, Armonk NY). Statistical analyses were performed both in the total sample and comparing two groups (non-LOBD vs. LOBD). Descriptive analyses were conducted by analyzing categorical variables' frequencies (*n*) and percentages (%). After analyzing the continuous variables for skewness, kurtosis, normality distribution through the Shapiro-Wilk test, and the equality of variances by Levene test, parametric or non-parametric statistical tests were used when appropriate. Normally distributed continuous variables were represented using the standard deviation (SD) or, if not normally distributed, the median and the confidence interval. Student's *t*-test for independent data and the non-parametric Mann-Whitney *U*-test for independent data were used, when appropriate, to compare primary outcome (i.e., TEMPS-M scores) and other secondary continuous variables between non-LOBD vs. LOBD group. The Chi-Square test was used to examine differences in the distribution of categorical variables between non-LOBD vs. LOBD group. Bivariate Pearson's correlations have been used to investigate potential relationships between TEMPS-M scores and other secondary continuous variables. Linear regression analysis was performed to investigate the associations between the age of illness onset (dependent variable) and TEMPS-M scores (independent variables); and between the age of illness onset (dependent variable) and MOCA scores (independent variables). While identifying whether specific predominant affective temperament, cognitive pattern and gender may be predictors of LOBD, we utilized binary logistic regression analyses and estimated the odds ratios along with the 95% confidence intervals (95% CI). All the analyses were two-sided with α of 0.05.

## Results

### Sociodemographic Characteristics of the Sample

Sociodemographic and clinical characteristics of the included subjects are summarized in Table 1. A total of 180 patients affected with BD-I, BD-II, and CYC were screened. Of these, a total of 78 patients were firstly recruited, according to their age at their first assessment. Twelve patients were deleted due to exclusion criteria. Within the final sample (*n* = 66), the mean age was 64.5 ± 9.1 years, with more representativeness by female participants (*n* = 43; 65.2%). Most of the sample (*n* = 35; 53.1%) declared to be in a stable relationship (married or cohabiting). The majority of the sample had a high school diploma (*n* = 26; 39.4%). At the time of clinical assessment, half of the participants declared to be retired (*n* = 33). Most participants reported a concomitant medical condition (*n* = 40; 60.6%), mainly cardiovascular diseases (*n* = 21; 31.8%). Most subjects denied current or previous alcohol use and/or substance use (*n* = 52; 78.8%) (Table 1).

**Table 1:** Baseline sociodemographic, clinical characteristics, pharmacological subcategories.

		**Total sample**		**Non-LOBD**		**LOBD**		***p*-value**
Number of recruited subjects		66		33 (50.0%)		33 (50.0%)		
Mean age (years ± standard deviation)		64.5 ± 9.1		61.4 ± 7.9		67.6 ± 9.2		0.005*
Age of onset (years ± standard deviation)		44.2 ± 21.2		24.4 ± 4.4		63.9 ± 9.3		<0.001*
		***n***	**%**	***n***	**%**	***n***	**%**	
**Gender**	Female	43	65.2	18	54.5	25	75.8	**$\chi_{\left(1\right)}^{2}$ = 3.270
	Male	23	34.8	15	45.5	8	24.2	*p* = 0.060
**Marital status**	Single	6	9.1	6	18.2	0	0	**$\chi_{\left(4\right)}^{2}$ = 13.280
	Cohabiting	4	6.1	1	3.0	3	9.1	***p*** **=** **0.010**
	Married	31	47.0	11	33.3	20	60.6	
	Separated/divorced	15	22.7	11	33.3	4	12.1	
	Widowed	10	15.2	4	12.1	6	18.2	
**Educational level**	Elementary education	13	19.7	3	9.1	10	30.3	**$\chi_{\left(3\right)}^{2}$ = 5.976
	Inferior middle license	19	28.8	10	30.3	9	27.3	*p* = 0.113
	Superior middle license	26	39.4	14	42.4	12	36.4	
	University degree	8	12.1	6	18.2	2	6.1	
**Profession**	Unoccupied	8	12.1	6	18.2	2	6.1	**$\chi_{\left(4\right)}^{2}$ = 13.097
	Part-time	3	4.5	3	9.1	0	0	***p*** **=** **0.011**
	Full-time	14	21.2	5	15.2	9	27.3	
	Retired	33	50.0	12	36.4	21	63.6	
	Unable to work	8	12.1	7	21.2	1	3.0	
**Comorbidities**	Existing	40	60.6	23	69.7	17	51.5	**$\chi_{\left(1\right)}^{2}$ = 2.285
	None	26	39.4	10	30.3	16	48.5	*p* = 0.131
*Cardiovascular*	Existing	21	31.8	12	36.4	9	27.3	**$\chi_{\left(1\right)}^{2}$ = 0.629
	None	45	68.2	21	63.6	24	72.7	*p* = 0.428
*Metabolic*	Existing	19	28.8	14	42.4	5	15.2	**$\chi_{\left(1\right)}^{2}$ = 5.987
	None	47	71.2	19	57.6	28	84.8	***p*** **=** **0.014**
*Endocrinological*	Existing	15	22.7	12	36.4	3	9.1	**$\chi_{\left(1\right)}^{2}$ = 6.988
	None	51	77.3	21	63.6	30	90.9	***p*** **=** **0.008**
*Osteoarticular*	Existing	10	15.2	5	15.2	5	15.2	**$\chi_{\left(1\right)}^{2}$ = 0.000
	None	56	84.8	28	84.8	28	84.8	*p* = 1.000
*Others*	Existing	33	50.0	18	54.5	15	45.5	**$\chi_{\left(1\right)}^{2}$ = 0.545
	None	33	50.0	15	45.5	18	54.5	*p* = 0.460
**Diagnosis**	Bipolar disorder I	39	59.1	30	90.9	9	27.3	**$\chi_{\left(2\right)}^{2}$ = 27.692
	Bipolar disorder II	26	39.4	3	9.1	23	69.7	***p*** **<** **0.001**
	Cyclothymic disorder	1	1.5	0	0	1	3.0	
**Current affective episode**	Depressive	33	50.0	12	36.4	21	63.6	**$\chi_{\left(3\right)}^{2}$ = 9.323
	Manic	19	28.8	15	45.5	4	12.1	***p*** **=** **0.025**
	Hypomanic	8	12.1	3	9.1	5	15.2	
	Euthymia	6	9.1	3	9.1	3	9.1	
**Specifiers**	Anxiety	12	18.2	4	12.1	8	24.2	**$\chi_{\left(4\right)}^{2}$ = 13.616
	Mixed characteristics	34	51.5	13	39.4	21	63.6	***p*** **=** **0.009**
	Psychotic characteristics	9	13.6	9	27.3	0	0	
	Atypical characteristics	10	15.2	6	18.2	4	12.1	
	Seasonal trend	1	1.5	1	3.0	0	0	
**Suicide attempts**	Positive history	11	16.7	3	9.1	8	24.2	**$\chi_{\left(1\right)}^{2}$ = 2.727
	Negative history	55	83.3	30	90.9	25	75.8	*p* = 0.099
**Self-harm**	Positive history	8	12.1	4	12.1	4	12.1	**$\chi_{\left(1\right)}^{2}$ = 0.000
	Negative history	58	87.9	29	87.9	29	87.9	*p* = 1.000
**Non-psychiatric drug therapy**	Immunosuppressants	1	1.5	1	3.0	0	0	
	Antihypertensives	24	36.4	13	39.4	11	33.3	**$\chi_{\left(5\right)}^{2}$ = 8.786
	L-Thyroxine	4	6.1	3	9.1	1	3.0	*p* = 0.186
	Antidiabetics	3	4.5	1	3.0	2	6.1	
	Antiplatelets	4	6.1	4	12.1	0	0	
	Statins	2	3.0	1	3.0	1	3.0	
	None	28	42.4	10	30.3	18	54.5	
**Antipsychotics**	Risperidone	1	1.5	1	3.0	0	0	**$\chi_{\left(5\right)}^{2}$ = 9.300
	Aripiprazole	12	18.2	10	30.3	2	6.1	*p* = 0.098
	Olanzapine	8	12.1	3	9.1	5	15.2	
	Quetiapine	20	30.3	8	24.2	12	36.4	
	Clozapine	1	1.5	1	3.0	0	0	
	None	24	36.4	10	30.3	14	42.4	
**Mood stabilizers**	Lithium	19	28.8	13	39.4	6	18.2	**$\chi_{\left(4\right)}^{2}$ = 6.261
	Valproate	15	22.7	8	24.2	7	21.2	*p* = 0.180
	Lamotrigine	4	6.1	1	3.0	3	9.1	
	Carbamazepine	2	3.0	0	0	2	6.1	
	None	26	39.4	11	33.3	15	45.5	
**Antidepressants**	SSRIs	21	31.8	8	24.2	13	39.4	**$\chi_{\left(6\right)}^{2}$ = 6.057
	SNRIs	3	4.5	1	3.0	2	6.1	*p* = 0.417
	Trazodone	5	7.6	2	6.1	3	9.1	
	Mirtazapine	1	1.5	0	0	1	3.0	
	Reboxetine	1	1.5	0	0	1	3.0	
	Vortioxetine	5	7.6	3	9.1	2	6.1	
	None	30	45.4	19	57.6	11	33.3	

*LOBD, Late Onset Bipolar Disorder; SSRIs, Selective Serotonin Reuptake Inhibitors; SNRIs, Serotonin and Norepinephrine Reuptake Inhibitors.*

^*^U Mann-Whitney-test.

^**^χ^2^-test. Bold values indicate a significance level.

### Psychopathological Characteristics

General psychopathological characteristics are summarized in Table 1. According to DSM-5 criteria (29), 39 subjects were diagnosed with BD-I (59.1%), 26 with BD-II (39.4%), and only one patient with a CYC (1.5%). At the time of their first assessment, most of the sample had a current depressive episode (*n* = 33; 50%), with mixed characteristics as the most prevalent specifier of the affective episode (*n* = 34; 51.5%). Most participants declared a negative history of suicide attempt (*n* = 55; 83.3%) or self-harm (*n* = 58; 87.9%). Table 1 also summarizes non-psychiatric and psychopharmacological therapy at the time of their first assessment. Predominant hyperthymic and depressive affective temperaments were found to be the most frequently represented in the total sample (Table 2). Table 2 summarizes mean scores within the total sample and both groups. The neurocognitive assessment showed a mean MOCA total score of 24.7 ± 3.2, with 56.1% of the sample with a MOCA total score indicative of MCI (Table 2).

**Table 2:** Psychopathological scores.

	**Total sample**		**Non-LOBD**		**LOBD**	
	***n***	**%**	***n***	**%**	***n***	**%**
**TEMPS-M**						
Depressive	17	25.8	7	21.2	10	30.3
Cyclothymic	8	12.1	4	12.1	4	12.1
Hyperthymic	26	39.4	17	51.5	9	27.3
Irritable	6	9.1	2	6.1	4	12.1
Anxious	9	13.6	3	9.1	6	18.2
	**Mean** **±** **SD**	**Mean** **±** **SD**	**Mean** **±** **SD**	***p*** -value*		
**TEMPS-M**						
Depressive	18.2 ± 7.3	16.2 ± 6.5	20.3 ± 7.5	**0.021**		
Cyclothymic	17.7 ± 7.1	16.7 ± 6.4	18.6 ± 7.8	0.375		
Hyperthymic	20.4 ± 7.2	21.0 ± 7.0	19.7 ± 7.6	0.422		
Irritable	15.8 ± 7.2	14.8 ± 6.7	16.9 ± 7.6	0.203		
Anxious	17.3 ± 6.9	14.1 ± 6.1	20.5 ± 6.3	**<0.001**		
Total	88.9 ± 21.7	82.7 ± 18.1	95.0 ± 23.5	0.063		
**CGI-BD**	4.3 ± 1.1	4.4 ± 1.1	4,3 ± 1.1	0.701		
**GAF**	52.5 ± 18.6	51.5 ± 18.3	53.4 ± 19.1	0.695		
**BPRS**	48.7 ±13.8	48.7 ± 14.5	48.7 ± 13.4	0.788		
**MOCA**	24.7 ± 3.2	24.1 ± 3.0	24.3 ± 3.4	0.334		
**HAM-D**	19.5 ± 10.1	16.0 ± 7.5	23.0 ± 11.2	**0.012**		
**GDS**	7.7 ± 4.2	5.9 ± 3.7	9.6 ± 3.8	**<0.001**		
**YMRS**	13.5 ± 9.8	15.3 ± 10.7	11.6 ± 8.6	0.256		
**MSRS**	8.7 ± 3.1	7.6 ± 3.2	9.8 ± 2.7	**0.005**		

*LOBD, Late Onset Bipolar Disorder; n, number; SD, standard deviation; TEMPS-M, Temperament Evaluation in Memphis, Pisa and San Diego; CGI-BD, Clinical Global Impression Severity Scale-Bipolar Disorder; GAF, General Assessment of Functioning; BPRS, Brief Psychiatric Rating Scale; MOCA, Montreal Cognitive Assessment; HAM-D_21_, Hamilton Depression Rating Scale-21 items; GDS, Geriatric Depression Scale; YMRS, Young Mania Rating Scale; MSRS, Mixed States Rating Scales.*

*Student's T-test referring to comparisons between non-LOBD vs. LOBD group. Bold values indicate a significance level.

### Comparison Between Non-LOBD and LOBD

The mean age of non-LOBD group is statistically significant lower than the LOBD group (61.4 ± 7.9 and 67.6 ± 9.2, respectively, *p* = 0.005). Only a not significantly statistical trend was observed in gender distribution between non-LOBD and LOBD group (54.5% of females in non-LOBD group vs. 75.8% in LOBD group; *p* = 0.07). LOBD subjects (*n* = 20; 15.5%) were mostly married compared to non-LOBD (*n* = 11; 33.3%) (*p* = 0.010). LOBD subjects (*n* = 21; 63.6%) were significantly more likely to be already retired, compared to non-LOBD (*n* = 12; 36.4%) (*p* = 0.011). Non-LOBD subjects declared significantly more endocrinological comorbidities (*p* = 0.008), metabolic comorbidities (*p* = 0.014), compared to LOBD subjects (Table 1).

Both subsamples were homogeneous for the cognitive pattern at MOCA, global functioning, and impairment at CGI-BD and GAF, as well as for general psychopathology (as measured by BPRS) and manic symptomatology (as measured by YMRS) (Table 2). Our non-LOBD subsample is mainly constituted by subjects diagnosed with BD-I (*n* = 30; 90.9%), whereas the LOBD subsample is mostly represented by subjects affected with BD-II (*p* <0.001; Table 1). At the time of their first assessment, most LOBD subjects displayed a depressive episode (*p* = 0.025) frequently associated with mixed features (*p* = 0.009), compared to non-LOBD subjects (Table 1). In this regard, statistically significant higher HAM-D scores (*p* = 0.012), GDS scores (*p* <0.001) and MSRS scores (*p* = 0.005) were found among LOBD subjects, compared to non-LOBD subjects at the time of their first assessment. Within the LOBD subsample, a positive linear correlation was found between GDS and HAM-D (*r* = 0.480), GDS and MSRS (*r* = 0.578), HAM-D and MSRS (*r* = 0.594).

Linear regression analysis demonstrated that depressive [*F*_(1, 64)_ = 4.307, *R*^2^ = 0.063, *p* = 0.042] and anxious temperaments [*F*_(1, 64)_ = 15.441, *R*^2^ = 0.194, *p* < 0.001] statistically significantly predicted age of BD onset. A positive correlation was found between depressive (*r* = 0.298) and anxious affective temperament (*r* = 0.419) and the age of illness onset. A logistic regression was performed to ascertain the effects of gender, MOCA and TEMPS-M on the likelihood of LOBD. The logistic regression model was statistically significant, $\chi_{\left(8\right)}^{2}$ = 22.879, *p* = 0.004. The model explained 39.1% (Nagelkerke *R*^2^) of the variance in LOBD and correctly classified 74.2% of cases. Subjects with higher scores at anxious temperament own an OR = 1.4 to exhibit LOBD [Exp(*B*) = 1.434; *B* = 0.361; *p* = 0.018].

## Discussion

Overall, our findings documented a statistically significant prevalence of predominant depressive and anxious affective temperaments in LOBD subjects compared to non-LOBD. Predominant anxious and depressive affective temperaments significantly predicted age of BD onset, with a positive correlation. In particular, subjects with higher scores at TEMPS-M anxious subscale were 1.43 times more likely to exhibit a later BD onset. The predominant hyperthymic temperament profile was more represented in the non-LOBD group, even though it was not found to have a clinical significance. This finding may be partially explained also by the low representativeness of subjects with a positive history of suicidal ideation and/or attempt and/or self-injury in our sample, as already documented in previous studies which demonstrated that a predominant hyperthymic affective temperament is overly protective against suicidal risk (39, 40). Furthermore, non-LOBD group was more represented by BD-I subjects who usually more likely display hyperthymic temperament (37, 41). In our study, only a gender trend was found with females more represented in the LOBD group, which may partially explain the higher prevalence of depressive and anxious temperaments in LOBD (14, 37, 42, 43). Our primary hypothesis was that specific predominant affective temperament profiles might be a clinical determinant that may influence BD's clinical expression, including the age of illness onset (13, 14, 43). The affective temperament may be defined as the basic affective predisposition to a level of activity, affective tone, or mood and their intensity, reactivity, and variability (43). Emily Kraepelin (44) firstly described four basic affective dispositions (depressive, manic, cyclothymic, and irritable) and proposed that the unbalance among those might be the main determinant of affective disorders (45). Akiskal et al. (43) formulated a psychopathological continuum between affective temperaments and affective disorders, introducing the concept of the bipolar spectrum and included also a fifth temperament (anxious). Affective temperaments are components of the spectrum of affective disorders, which encompasses recurrent depressive disorder, dysthymia, depressive, hyperthymic and cyclothymic temperaments, mixed affective states, hypomania, and BD-I and BD-II (43). At this regard, further authors supported the hypothesis that the hyperthymic and depressive temperaments are more related to the more “classic” bipolar picture, while cyclothymic, anxious and irritable temperaments are related to more complex pictures and might predict poor response to treatment, violent and suicidal behaviour and high medical comorbidity (14, 46–48).

Moreover, although both subsamples appear to be homogeneous regarding global functioning, general psychopathology, and manic symptomatology, LOBD individuals significantly displayed a more severe depressive symptomatology and mixed states, as previously documented (45, 49, 50). However, one could argue that this depressive trend may be influenced by the most representativeness of female subjects in our sample and by the most frequent prevalence of BD-II patients in the LOBD group. MSRS scores documented that LOBD individuals showed significantly higher levels of mixed states over the previous months, by underlining how mixed states may not be only limited to the current depressive episode (15). Furthermore, our findings appear to confirm a gender-based trend in the onset of LOBD, in line with existing literature (50, 51). Female gender may in fact constitute a predictive factor for LOBD, as much as it is for a higher prevalence of BD type II, hypomania, and mixed episodes (12, 52). Hormonal and environmental changes such as menopause have been identified as possible triggers of LOBD (53–56). Similarly, mixed episodes in LOBD subjects with depressive affective temperaments may be explained by a gender-based effect, as already documented in previous studies (12, 14).

Regarding global functioning and the presence of protective factors of illness onset, LOBD were statistically more frequently retired and in a stable affective relationship. These findings may partially support, on one hand, the hypothesis that LOBD patients may have displayed an attenuated/subsyndromal manifestation over their life which did not manifest in full-blown psychopathology thanks to the presence of family and social protective factors. On the other hand, one could argue that the higher prevalence of retired LOBD individuals may imply that retirement could represent a predisposing risk factor in those individuals with a vulnerability or a previous attenuated form. However, the limitations of our cross-sectional study may not have allowed us to completely explore this hypothesis, which should be better deepened in further larger longitudinal studies.

Finally, our findings did not report any significant differences in cognitive patterns between non-LOBD and LOBD, being MOCA scores homogeneous between two groups, in line with previous studies (57, 58). However, being our sample mainly constituted by patients scoring above 20 at MOCA, in order to avoid confounding biases, one could argue that our findings might not be generalizable to geriatric BD population, as already investigated by Tsai et al. (59) and Azorin et al. (60). Furthermore, the role of cerebrovascular disease in the pathophysiology of mood and cognitive symptoms in LOBD has been an increasingly critical concern (61). However, our findings did not report a higher prevalence of cardiovascular diseases in LOBD. Therefore, our exploratory hypothesis that a MCI may more likely accompany a LOBD was not sufficiently confirmed. However, further larger longitudinal studies should be conducted to explore this hypothesis, also including brain structural MRI studies, with volumetric analyses of white matter hyperintensities and gray matter volume, Diffusion Tensor Imaging (DTI) studies, and a full neurocognitive assessment (16, 62). In line with existing literature, our findings reported a significantly higher prevalence of medical comorbidities in the non-LOBD group, particularly metabolic and endocrinological diseases (63, 64). Indeed, BD has been conceptualized as a multi-system disease rather than a brain-specific disease (65, 66). Moreover, it is arguable that the broader use of antipsychotics and mood stabilizers over the lifespan may have predisposed non-LOBD individuals to a higher occurrence of metabolic and endocrinological side effects (67–70). Moreover, one could argue that clinical course and illness duration may become significant variables that could influence the adoption of unhealthy lifestyles, which in turn may determine significant determinants of medical comorbidity onset in non-LOBD (7, 71).

Despite the promising preliminary findings, our study presents several limitations. Firstly, this is a preliminary and exploratory study in real-world practice, recruiting subjects in a naturalistic setting retrospectively. Secondly, the study has a relatively small sample size without a healthy control group of elderly individuals. In addition, there is a lack of a further stratification for the comparison group which may raise some generalizability of the results concerns. Thirdly, in our sample there is poor representativeness of BD subjects with suicidal ideation, self-harming, and/or suicide attempts, even though this may be partially explained in the view of the fact that hyperthymic temperament was the most represented in the sample. Therefore, we did not collect data on affective temperamental profiles among LOBD vs. non-LOBD with a suicidality pattern. Moreover, our sample is not representative of dual diagnosis BD patients who may display another temperament profile in the LOBD group compared to non-LOBD. In addition, our sample has been mainly assessed during a depressive bipolar episode, as during this phase the individual displays more insight and more likely agrees to be assessed. Furthermore, even if the severity of psychopathology and BD-specific symptom domains were mainly analyzed using clinician-rated rating scales, affective temperament was assessed by a self-rated scale with potential biases due to the intrinsic nature of self-rating scales. In addition, being our sample mainly represented by BD outpatients, our findings might not be completely generalizable and, hence, further studies including also inpatients are needed to confirm our preliminary findings. Furthermore, as our sample selected only patients scoring above 20 at MOCA to avoid confounding biases, one could argue that the most severe LOBD cases might get excluded (i.e., Berkson's bias) and this might potentially be an issue undermining the overall generalizability of the findings. Finally, our study did not collect BD-specific biomarkers and/or neuroimaging data or assess patients with a full neurocognitive battery, as EMRs retrospectively collected all data.

A critical issue was also establishing which cut-off age was needed to discriminate between non-LOBD and LOBD. Although there is still uncertainty and contrasting ideas among scientific communities, our study was built by considering the threshold age proposed by the ISBD Task Force (7). In fact, the ISBD Task Force proposed defining LOBD those individuals who displayed a first manic/hypomanic episode at >50 years and individuals who have had prior depressive episodes without manic/hypomanic episodes until age>50 years, as already suggested by other researchers (7, 24, 25, 27). However, our inclusion criteria may have determined potential recruitment biases. In fact, other clinicians suggested anticipating the cut-off at 40 years to allow a better interpretation of those cases arising at an unusual age, between an EOBD, an intermediate/conventional BD onset, and a LOBD (72).

In conclusion, there is not enough evidence to demonstrate whether LOBD and non-LOBD may display different clinical characterization, also in terms of treatment strategies and outcomes. The increased number of individuals with an old age BD already overburdened healthcare systems which should adapt to this demographic change. Current psychogeriatric research underscores the importance of a lifespan perspective in research and clinical practice, particularly in BD clinical courses and age of onset. It may be stated that attenuated/subsyndromal symptomatology may partially explain the later onset of BD among those individuals with predisposing factors and “bipolarity” vulnerability (73), including identifying specific markers for age of BD onset such as specific predominant affective temperamental profiles which could serve to predict clinical outcomes and treatment targets. Furthermore, there is the need to better understand whether sub-diagnostic hypomanic symptoms lifetime (potentially leading to overdiagnosis of major depressive disorder and dysthymia) may partially explain a later BD diagnosis (74–76). Specific predominant affective temperaments might constitute vulnerability factors, as well as clinical picture and illness course modifiers.

Further research should address specific markers of neuroinflammation, oxidative stress, and mitochondrial dysfunction, which could help characterize pathways supportive of a model of progressive deterioration that may predispose to the later onset of attenuated/subsyndromal BD during the lifetime. Further studies examining structural MRI changes over the lifespan may be a more useful approach to determining evidence for LOBD as a neuroprogressive disorder or a distinct subtype of BD. Moreover, further longitudinal studies should evaluate the differential expected trajectory and prognosis between non-LOBD and LOBD in elderly patients, including identifying lifetime sub-diagnostic hypomanic symptoms. In particular, it may be investigated whether specific psychopharmacological treatment (such as lithium) may determine a neuroprotective effect and whether modifying specific lifestyle factors may differently impact long-term outcomes and later onset of those attenuated bipolar spectrum forms, including whether a partial adherence, the number (and type) of affective episodes and number of recurrent episodes occurring in the lifespan might modify psychopathological trajectory and, hence, influence BD onset. Early identifying predictors of LOBD may potentially help clinicians to better manage treatment strategies in elderly.

## Data Availability Statement

The raw data supporting the conclusions of this article will be made available by the authors, without undue reservation.

## Ethics Statement

The local Institutional Review Board (IRB) approved the study, in accordance with the local legislation and institutional requirements. The patients/participants provided their informed consent to participate in this study.

## Author Contributions

LO and UV: conceptualization and methodology. LO, GM, and ST: formal analysis and writing—original draft preparation. DR, MF, LO, and ST: investigation. LO, ST, and MF: data curation. LO, AT, VS, and UV: writing—review and editing. VS: visualization. AT and UV: supervision. All authors have read and agreed to the published version of the manuscript.

## Conflict of Interest

The authors declare that the research was conducted in the absence of any commercial or financial relationships that could be construed as a potential conflict of interest.

## Publisher's Note

All claims expressed in this article are solely those of the authors and do not necessarily represent those of their affiliated organizations, or those of the publisher, the editors and the reviewers. Any product that may be evaluated in this article, or claim that may be made by its manufacturer, is not guaranteed or endorsed by the publisher.

## References

[1] Brown GC. Living too long: the current focus of medical research on increasing the quantity, rather than the quality, of life is damaging our health and harming the economy. EMBO Rep. (2015) 16:137–41. 10.15252/embr.201439518 25525070 PMC4328740

[2] Aburto JM Villavicencio F Basellini U Kjærgaard S Vaupel JW. Dynamics of life expectancy and life span equality. Proc Natl Acad Sci USA. (2020) 117:5250–9. 10.1073/pnas.1915884117 32094193 PMC7071894

[3] World Health Organization. Ageing and Health. (2021). Available online at: https://www.who.int/news-room/fact-sheets/detail/ageing-and-health (accessed November 30, 2021).

[4] Beard JR Officer A de Carvalho Sadana IA Pot R Michel AM. The world report on ageing and health: a policy framework for healthy ageing. Lancet. (2016) 387, 2145–2154. 10.1016/S0140-6736(15)00516-4 26520231 PMC4848186

[5] Sims J. Healthy aging. Aust Fam Phys. (2017) 46:1–2. 28189127

[6] Crestani C Masotti V Corradi N Schirripa ML Cecchi R. Suicide in the elderly: a 37-years retrospective study. Acta Biomed. (2019) 90:68–76. 10.23750/abm.v90i1.6312 30889157 PMC6502164

[7] Sajatovic M Strejilevich SA Gildengers AG Dols A Al Jurdi RK Forester BP. report on older-age bipolar disorder from the International Society for Bipolar Disorders Task Force. Bipolar Disord. (2015) 17:689–704. 10.1111/bdi.12331 26384588 PMC4623878

[8] Almeida OP Hankey GJ Yeap BB Golledge J Flicker L. Older men with bipolar disorder: clinical associations with early and late onset illness. Int J Geriatr Psychiatr. (2018) 33:1613–9. 10.1002/gps.4957 30015397

[9] McIntyre RS Berk M Brietzke E Goldstein BI López-Jaramillo C Kessing LV. Bipolar disorders. Lancet. (2020) 396:1841–56. 10.1016/S0140-6736(20)31544-0 33278937

[10] Shobassy A. Elderly bipolar disorder. Curr Psychiatry Rep. (2021) 23:5. 10.1007/s11920-020-01216-6 33404961

[11] Dols A. Older age bipolar disorder. Clin Geriatr Med. (2020) 36:281–6. 10.1016/j.cger.2019.11.008 32222302

[12] Cassano GB Akiskal HS Savino M Musetti L Perugi G. Proposed subtypes of bipolar II and related disorders: with hypomanic episodes (or cyclothymia) and with hyperthymic temperament. J Affect Disord. (1992) 26:127–40. 10.1016/0165-0327(92)90044-7 1447430

[13] Mendlowicz MV Jean-Louis G Kelsoe JR Akiskal HSA. comparison of recovered bipolar patients, healthy relatives of bipolar probands, and normal controls using the short TEMPS-A. J Affect Disord. (2005) 85:147–51. 10.1016/j.jad.2004.01.012 15780685

[14] Fountoulakis KN Gonda X Koufaki I Hyphantis T Cloninger CR. The role of temperament in the etiopathogenesis of bipolar spectrum illness. Harv Rev Psychiatry. (2016) 24:36–52. 10.1097/HRP.0000000000000077 26735322

[15] Schürhoff F Bellivier F Jouvent R Mouren-Siméoni MC Bouvard M Allilaire JF. Early and late onset bipolar disorders: two different forms of manic-depressive illness? J Affect Disord. (2000) 58:215–21. 10.1016/S0165-0327(99)00111-1 10802130

[16] Doty TJ Payne ME Steffens DC Beyer JL Krishnan KR LaBar KS. Age-dependent reduction of amygdala volume in bipolar disorder. Psychiatry Res. (2008) 163:84–94. 10.1016/j.pscychresns.2007.08.003 18407469 PMC2483539

[17] Judd LL Akiskal HS Schettler PJ Coryell W Endicott J Maser JD. prospective investigation of the natural history of the long-term weekly symptomatic status of bipolar II disorder. Arch Gen Psychiatry. (2003) 60:261–9. 10.1001/archpsyc.60.3.261 12622659

[18] Correll CU Penzner JB Lencz T Auther A Smith CW Malhotra AK. Early identification and high-risk strategies for bipolar disorder. Bipolar Disord. (2007) 9:324–238. 10.1111/j.1399-5618.2007.00487.x 17547579

[19] Strejilevich SA Martino DJ Murru A Teitelbaum J Fassi G Marengo E. Mood instability and functional recovery in bipolar disorders. Acta Psychiatr Scand. (2013) 128:194–202. 10.1111/acps.12065 23331090

[20] Strejilevich S Szmulewicz A Igoa A Marengo E Caravotta P Martino D. Episodic density, subsyndromic symptoms, and mood instability in late-life bipolar disorders: a 5-year follow-up study. Int J Geriatr Psychiatry. (2019) 34:950–6. 10.1002/gps.5094 30864181

[21] Szmulewicz AG Martino DJ Strejilevich SA. Characterization of mood instability through bipolar disorders: a cluster-analytic approach using weekly prospective life-chart methodology. Eur Psychiatry. (2019) 57:52–7. 10.1016/j.eurpsy.2018.10.003 30677548

[22] Schulman KI Herrmann N. Bipolar disorder in old age. In: Marneros A, Angst J, editors. Bipolar Disorders: 100 Years After Manic-depressive Insanity. Dordrecht: Kluwer Academic Publishers (2000). p. 153–74.

[23] Moorhead SR Young AH. Evidence for a late onset bipolar-I disorder sub-group from 50 years. J Affect Disord. (2003) 73:271–7. 10.1016/S0165-0327(01)00476-1 12547296

[24] Depp CA Jeste DV. Bipolar disorder in older adults: a critical review. Bipolar Disord. (2004) 6:343–67. 10.1111/j.1399-5618.2004.00139.x 15383127

[25] Vasudev A Thomas A. ‘Bipolar disorder' in the elderly: what's in a name? Maturitas. (2010) 66:231–5. 10.1016/j.maturitas.2010.02.013 20307944

[26] Stubbs B Vancampfort D Solmi M Veronese N Fornaro M. How common is bipolar disorder in general primary care attendees? A systematic review and meta-analysis investigating prevalence determined according to structured clinical assessments. Aust N Z J Psychiatry. (2016) 50:631–9. 10.1177/0004867415623857 26764372

[27] Ljubic N Ueberberg B Grunze H Assion HJ. Treatment of bipolar disorders in older adults: a review. Ann Gen Psychiatry. (2021) 20:45. 10.1186/s12991-021-00367-x 34548077 PMC8456640

[28] American Psychiatric Association (APA). Statistical and Diagnostical Manual of Mental Disorders. APA Press (2013).

[29] Nasreddine ZS Phillips NA Bédirian V Charbonneau S Whitehead V Collin I. The Montreal Cognitive Assessment, MoCA: a brief screening tool for mild cognitive impairment. J Am Geriatr Soc. (2005) 53:695–9. 10.1111/j.1532-5415.2005.53221.x 15817019

[30] Spearing MK Post RM Leverich GS Brandt D Nolen W. Modification of the clinical global impressions (CGI) scale for use in bipolar illness (BP): the CGI-BP. Psychiatry Res. (1997) 73:159–71. 10.1016/S0165-1781(97)00123-6 9481807

[31] Endicott J Spitzer RL. The global assessment scale. A procedure for measuring overall severity of psychiatric disturbance. Arch Gen Psychiatry. (1976) 33:766. 10.1001/archpsyc.1976.01770060086012 938196

[32] Ventura J Green M. Training and quality assurance with the Brief Psychiatric Rating Scale: “the drift busters”. Int J Method Psychiat Res. (1993) 3:221.

[33] Hamilton M. A rating scale for depression. J Neurol Neurosurg Psychiat. (1960) 23:56. 10.1136/jnnp.23.1.56 14399272 PMC495331

[34] Sheikh JI Yesavage JA. Geriatric depression scale (GDS): recent evidence and development of a shorter version. Clin Gerontol J Aging Mental Health. (1986) 5:165–73. 10.1300/J018v05n01_0920629580

[35] Young RC Biggs JTA. rating scale for mania: reliability, validity and sensitivity. Br J Psychiat. (1978) 133:429. 10.1192/bjp.133.5.429 728692

[36] Tavormina G. Treating the bipolar spectrum mixed states: a new rating scale to diagnose them. Psychiatr Danub. (2014) 26:6–9. 25413502

[37] Fico G Luciano M Sampogna G Zinno F Steardo L Jr., Perugi G, et al. Validation of the brief TEMPS- M temperament questionnaire in a clinical Italian sample of bipolar and cyclothymic patients. J Affective Dis. (2020) 260:548–462. 10.1016/j.jad.2019.09.034 31539680

[38] Akiskal HS. Toward a temperament-based approach to depression: implications for neurobiologic research. Adv Biochem Psychopharmacol. (1995) 49:99–112. 7653337

[39] Vázquez GH Gonda X Zaratiegui R Lorenzo LS Akiskal K Akiskal HS. Hyperthymic temperament may protect against suicidal ideation. J Affect Disord. (2010) 127:38–42. 10.1016/j.jad.2010.04.015 20466435

[40] Janiri D De Rossi P Kotzalidis GD Girardi P Koukopoulos AE. Psychopathological characteristics and adverse childhood events are differentially associated with suicidal ideation and suicidal acts in mood disorders. Eur Psychiatry. (2018) 53:31–6. 10.1016/j.eurpsy.2018.05.009 29879623

[41] Perugi G Cesari D Vannucchi G Maccariello G Barbuti M Bartolomeis D. of affective temperaments on clinical and functional outcome of Bipolar I patients that initiated or changed pharmacological treatment for mania. Psychiatry Res. (2018) 261:473–80. 10.1016/j.psychres.2018.01.035 29360052

[42] Perugi G Musetti L Simonini E Piagentini F Cassano GB Akiskal HS. Gender-mediated clinical features of depressive illness. The importance of temperamental differences. Br J Psychiatry. (1990) 157:835–41. 10.1192/bjp.157.6.835 2289093

[43] Akiskal HS Akiskal KK Haykal RF Manning JS Connoe PD. TEMPS-A: progress towards validation of a self-rated clinical version of the Temperament Evaluation of the Memphis, Pisa, Paris, and San Diego Auto- questionnaire. J Affect Disord. (2005) 85:3–16. 10.1016/j.jad.2004.12.001 15780671

[44] Kraepelin E. Manic-depressive Insanity and Paranoia. Edinburgh: ES Livingstone (1921).

[45] Akiskal HS Pinto O. Soft Bipolar spectrum: footnotes to Kraepelin on the interface of hypomania, temperament and depression. Kluwer Acad. (2000) 2:37–62. 10.1007/0-306-47521-9_2

[46] Mazzarini L Pacchiarotti I Colom F Sani G Kotzalidis GD Rosa AR. polarity and temperament in bipolar and unipolar affective disorders. J Affect Disord. (2009) 119:28–33. 10.1016/j.jad.2009.03.016 19346002

[47] Azorin JM Adida M Belzeaux R. Predominant polarity in bipolar disorders: further evidence for the role of affective temperaments. J Affect Disord. (2015) 182:57–63. 10.1016/j.jad.2015.04.037 25973784

[48] Koufaki I Polizoidou V Fountoulakis KN. The concept of temperament and its contribution to the understanding of the bipolar spectrum. Psychiatriki. (2017) 28:142–55. 10.22365/jpsych.2017.282.142 28686561

[49] Bryant C. Anxiety and depression in old age: challenges in recognition and diagnosis. Int Psychoger. (2010) 22:511–3. 10.1017/S1041610209991785 20122303

[50] Garcia-Lopez A Ezquiaga E De Dios C Agud JL. Depressive symptoms in early and late-onset older bipolar patients compared with younger ones. Int J Geriatr Psychiatry. (2017) 32:201–7. 10.1002/gps.4465 27017999

[51] Goodwin FK Jamison KR. Manic-Depressive Illness: Bipolar Disorders and Recurrent Depression. 2nd ed. New York, NY: Oxford University Press (2007).

[52] Kessing LV. The prevalence of mixed episodes during the course of illness in bipolar disorder. Acta Psychiatr Scand. (2008) 117:216–24. 10.1111/j.1600-0447.2007.01131.x 18241307

[53] Ishimaru-Tseng TV. Evaluation of late onset bipolar illness during menopause. Hawaii Med J. (2000) 59:51–3. 10800252

[54] Hu LY Shen CC Hung JH Chen PM Wen CH Chiang YY. Risk of psychiatric disorders following symptomatic menopausal transition: a nationwide population-based retrospective cohort study. Medicine (Baltimore). (2016) 95:e2800. 10.1097/MD.0000000000002800 26871843 PMC4753939

[55] Chen LC Yang AC Su TP Bai YM Li CT Chang WH. Symptomatic menopausal transition and subsequent bipolar disorder among midlife women with major depression: a nationwide longitudinal study. Arch Womens Ment Health. (2017) 20:463–8. 10.1007/s00737-017-0725-x 28429098

[56] Perich TA Roberts G Frankland A Sinbandhit C Meade T Austin MP. Clinical characteristics of women with reproductive cycle-associated bipolar disorder symptoms. Aust N Z J Psychiatry. (2017) 51:161–7. 10.1177/0004867416670015 27687774

[57] Radanovic M Nunes PV Forlenza OV Braga Ladeira R Gattaz WF. Cognitive-linguistic deficits in euthymic elderly patients with bipolar disorder. J Affect Disord. (2013) 5:691–4. 10.1016/j.jad.2013.04.035 23764386

[58] Velosa J Delgado A Finger E Berk M Kapczinski F de Azevedo Cardoso T. Risk of dementia in bipolar disorder and the interplay of lithium: a systematic review and meta-analyses. Acta Psychiatr Scand. (2020) 141:510–21. 10.1111/acps.13153 31954065

[59] Tsai SY Lee HC Chen CC Huang YL. Cognitive impairment in later life in patients with early-onset bipolar disorder. Bipolar Disord. (2007) 9:868–75. 10.1111/j.1399-5618.2007.00498.x 18076536

[60] Azorin JM Kaladjian A Adida M Fakra E. Late-onset bipolar illness: the geriatric bipolar type VI. CNS Neurosci Ther. (2012) 18:208–13. 10.1111/j.1755-5949.2011.00255.x 22070456 PMC6493355

[61] Subramaniam H Dennis MS Byrne EJ. The role of vascular risk factors in late onset bipolar disorder. Int J Geriatr Psychiatry. (2007) 22:733–7. 10.1002/gps.1730 17146839

[62] Montejo L Jiménez E Solé B Murru A Arbelo N Benabarre A. Identifying neurocognitive heterogeneity in older adults with bipolar disorder: a cluster analysis. J Affect Disord. (2021) 298:522–31. 10.1016/j.jad.2021.11.028 34788686

[63] Tsai SY Kuo CJ Chung KH Huang YL Lee HC Chen CC. Cognitive dysfunction and medical morbidity in elderly outpatients with bipolar disorder. Am J Geriatr Psychiatry. (2009) 17:1004–11. 10.1097/JGP.0b013e3181b7ef2a 20104057

[64] Lala SV Sajatovic M. Medical and psychiatric comorbidities among elderly individuals with bipolar disorder: a literature review. J Geriatr Psychiatry Neurol. (2012) 25:20–5. 10.1177/0891988712436683 22467842

[65] Leboyer M Kupfer DJ. Bipolar disorder: new perspectives in health care and prevention. J Clin Psychiatry. (2010) 71:1689–95. 10.4088/JCP.10m06347yel 21190640 PMC3317891

[66] Salvi V Barone-Adesi F D'Ambrosio V Albert U Maina G. High H1-affinity antidepressants and risk of metabolic syndrome in bipolar disorder. Psychopharmacology (Berl). (2016) 233:49–56. 10.1007/s00213-015-4085-9 26407602

[67] Orsolini L Tomasetti C Valchera A Vecchiotti R Matarazzo I Vellante F. update of safety of clinically used atypical antipsychotics. Expert Opin Drug Saf. (2016) 15:1329–47. 10.1080/14740338.2016.1201475 27347638

[68] Howell S Yarovova E Khwanda A Rosen SD. Cardiovascular effects of psychotic illnesses and antipsychotic therapy. Heart. (2019) 105:1852–9. 10.1136/heartjnl-2017-312107 31439658

[69] Salvi V Aguglia A Barone-Adesi F Bianchi D Donfrancesco C Dragogna F. Cardiovascular risk in patients with severe mental illness in Italy. Eur Psychiatry. (2020) 63:e96. 10.1192/j.eurpsy.2020.94 33100262 PMC7681153

[70] Orsolini L Pompili S Volpe U. The ‘collateral side' of mood stabilizers: safety and evidence-based strategies for managing side effects. Expert Opin Drug Saf. (2020) 19:1461–95. 10.1080/14740338.2020.1820984 32893696

[71] Beunders AJM Kok AAL Kosmas PC Beekman ATF Sonnenberg CM Schouws SNTM. Physical comorbidity in Older-Age Bipolar Disorder (OABD) compared to the general population - a 3-year longitudinal prospective cohort study. J Affect Disord. (2021) 288:83–91. 10.1016/j.jad.2021.03.057 33845328

[72] Nivoli AM Murru A Pacchiarotti I. Bipolar disorder in the elderly: a cohort study comparing older and younger patients. Acta Psychiatr Scand. (2014) 130:364–73. 10.1111/acps.12272 24702648

[73] Strakowski SM Fleck DE Maj M. Broadening the diagnosis of bipolar disorder: benefits vs. risks. World Psychiatry. (2011) 10:181–6. 10.1002/j.2051-5545.2011.tb00046.x 21991268 PMC3188763

[74] Angst J Gamma A Lewinsohn P. The evolving epidemiology of bipolar disorder. World Psychiatry. (2002) 1:146–8. 16946835 PMC1489851

[75] Chen PD Sajatovic RS. Update on the epidemiology, diagnosis, and treatment of mania in older-age bipolar disorder. Curr Psychiatry Rep. (2017) 19:46. 10.1007/s11920-017-0804-8 28647815

[76] NICE National Institute for Health and Care Excellence. Bipolar Disorders: The Assessment and Management of Bipolar Disorder in Adults, Children and Young People in Primary and Secondary Care. London: NICE National Institute for Health and Care Excellence (2014).

